# Comparing the Composition of Small RNA in Blood Plasma Fractions Containing Exomeres and Supermeres

**DOI:** 10.3390/ijms27209268

**Published:** 2026-10-19

**Authors:** Aleksei Y. Aronov, Alexander A. Artamonov, Sergey A. Chekrigin, Evgeniy A. Grigoryev, Olga S. Vezo, Ruslan I. Glushakov, Kirill A. Kondratov

**Affiliations:** 1 Saint-Petersburg State University, 199034 St. Petersburg, Russia; aronov.aleksej@bk.ru (A.Y.A.); grigoryev_egeniy@mail.ru (E.A.G.);; 2 S. M. Kirov Military Medical Academy, 194044 St. Petersburg, Russiaglushakoffruslan@yandex.ru (R.I.G.); 3 Institute of Experimental Medicine, 197022 St. Petersburg, Russia

**Keywords:** exomeres, supermeres, sRNA, NGS, miRNA, piRNA, differential ultracentrifugation, small RNA patterns

## Abstract

It is known that small RNAs contained within extracellular vesicles can serve as agents of intercellular signaling. However, recent studies have shown that these molecules are carried not only by vesicles but also by smaller, non-membranous particles known as exomeres and supermeres. At the same time, the specific repertoire of small RNAs in these particles, as well as the extent to which the RNA profiles of exomeres and supermeres in human blood plasma differ—remains insufficiently studied. The aim of this study was to compare the small RNA patterns in blood plasma fractions containing these particles. Extracellular vesicles were first pelleted from platelet-poor plasma via ultracentrifugation (170,000× *g*, 4 h); subsequently, a fraction containing exomeres (FCE; pellet from 170,000× *g*, 20 h centrifugation) and a fraction containing supermeres (FCS; pellet from 370,000× *g*, 20 h centrifugation) were sequentially isolated. Subsequently, the small RNA composition was analyzed (FCE from eight donors and FCS from fourteen donors). It was found that the FCE and FCS fractions differed significantly in their non-coding RNA composition. Compared to FCE, FCS showed a significantly higher GC content in the small RNA fraction, as well as higher proportions of rRNA, tRNA, snoRNA, and snRNA, and lower proportions of miRNA, piRNA, lncRNA, and scaRNA. Analysis of the sequenced fragments length distribution revealed that, compared to FCE, the FCS sample contained a higher proportion of sequences shorter than 20 nucleotides (17–20 nt) and longer than 24 nucleotides (24–50 nt). A comparison of the major sequences showed that *miRNA-451a* accounts for approximately 50% of the total miRNA in FCE and 25% in FCS, while *piRNA-45195* constitutes about 50% of the total in both FCS and FCE. A comparison of the levels of various miRNAs in the two fractions showed that the levels of 184 miRNAs were significantly higher (*p*-adj < 0.05, fold change > 2) in the FCE fraction, while 3 miRNAs were significantly higher in the FCS fraction. Additionally, the levels of 13 piRNAs were significantly higher in FCE, and 34 in FCS. Thus, despite the fact that the patterns of the most abundant small RNAs in these fractions are quite similar, FCS and FCE most likely exhibit different signaling functions.

## 1. Introduction

Intercellular communication is traditionally viewed as a process mediated by individual secreted molecules. However, in recent decades, it has become evident that the transfer of biological information is also carried out by complex multimolecular assemblies. Extracellular vesicles play a key role in this process. In addition to vesicles, two fundamentally new classes of membrane-less extracellular nanoparticles—exomeres and supermeres—have recently been discovered and characterized [1]. It should be noted that there are currently no sufficiently clear molecular criteria to unequivocally distinguish between these two pools of particles, even in the most important human physiological fluids. At the same time, studies show that these nanoparticles carry sets of proteins, lipids, and nucleic acids that differ fundamentally from the contents of classical extracellular vesicles [2]. The functions of exomeres and supermeres are being actively studied. It has been shown that they are capable of transmitting signals between cells. For example, exomeres carrying the protein amphiregulin can activate the epidermal growth factor receptor signaling pathway in target cells [3]. In turn, supermeres demonstrate an even broader spectrum of activity: they are capable of transferring resistance to chemotherapeutic drugs, crossing the blood–brain barrier, and inducing metabolic changes in recipient cells [4]. RNA transport appears to be a crucial aspect of the signaling function of these particles. It has been demonstrated that both exomeres and supermeres contain RNA, with a significant proportion of extracellular RNA associated specifically with them rather than with vesicles [2]. Analysis of the RNA types present in exomeres and supermeres revealed that, in addition to miRNA, they also contain fragments of small nuclear RNA (snRNA), ribosomal RNA (rRNA), and other types of RNA [4]. The nucleic acid composition of exomeres and supermeres is being actively investigated, and it can vary significantly depending on the method used to culture the donor cells [2]. Most existing studies focus on analyzing the composition of these particles derived from cell culture media, particularly those from tumor cells [2,5]. However, despite growing interest, the small RNA composition of human plasma supermeres and exomeres remains poorly characterized, whereas the RNA composition of plasma extracellular vesicles has long been well-defined [6]. The systematic analysis of small RNAs from blood plasma-derived exomeres and supermeres remains a novel and relevant challenge. Little is known not only about which small RNAs are most abundant in blood plasma exomeres and supermeres, but also about the specific RNA profiles that distinguish these two groups of particles. The aim of our study was to analyze the small RNA pool within human blood plasma exomeres and supermeres.

## 2. Results

**Figure 1:**
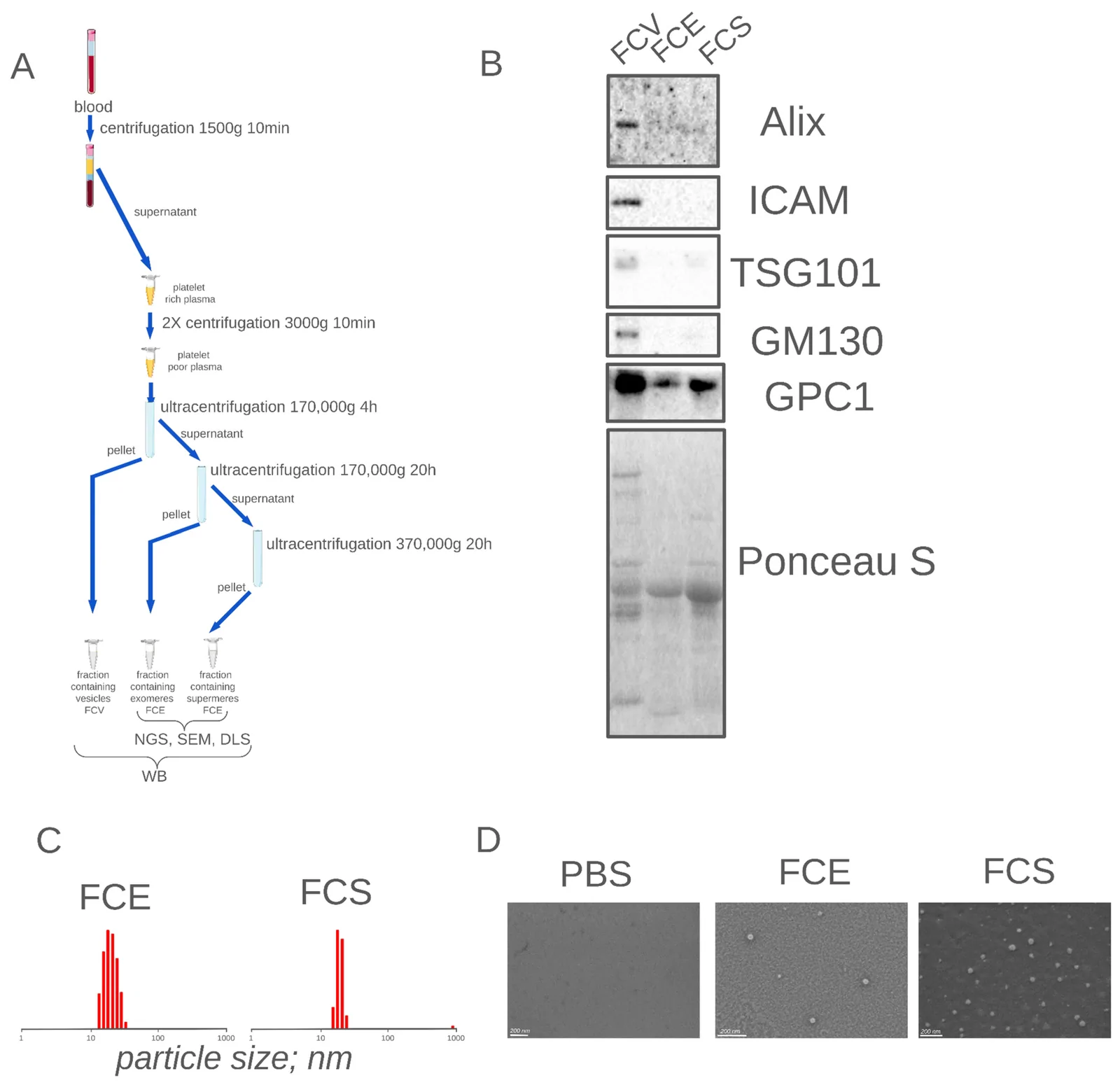
Obtaining, preparing and general characterization of human plasma fractions containing vesicles (FCVs), exomeres (FCEs), and supermeres (FCSs). (**A**) Scheme of obtaining plasma fractions. (**B**) Comparison of GM130, Alix, TSG101, Ago2, and GPC1 protein levels in FCV, FCE, and FCS by Western blotting. (**C**) Comparison of particle sizes in FCF and FCS fractions by dynamic light scattering. (**D**) Particle morphology in FCF, FCS, and control buffer (PBS).

Blood plasma fractions were isolated using differential ultracentrifugation (Figure 1A). Following the isolation of fractions containing exomeres and supermeres, particle sizes within these fractions were assessed using dynamic light scattering; the results are presented in Figure 1C. As shown in the figure, the particle sizes in both fractions were approximately 18 nm. The study also examined particle morphology using low-voltage scanning electron microscopy. As shown in Figure 1D, small particles (5–40 nm)—absent from the control buffer—were observed in the fractions. Thus, particles with the corresponding morphology and size were present in the isolated fractions. Additionally, the levels of vesicle marker proteins (ICAM, Alix, TSG101, GM130) and supermere markers (GPC1) were compared across the fractions (Figure 1B). To demonstrate uniform protein loading across the lanes, the membrane was stained with the nonspecific dye Ponceau S (shown below). As seen in the figure, vesicle markers were present only in FCV; however, GPC1 was detected not only in FCV but also in FCE and FCS. It is known that in addition to supermeres [4], this protein can be part of extracellular vesicles [7]. It should also be noted that it is not yet fully established which proteins can be considered unique markers of exomeres and supermeres. For example, Ago2 has been detected both in extracellular vesicles [8] and in exomeres [9], while ACE2 has been detected in extracellular vesicles [10] as well as in supermeres [4]. Therefore, FCE and FCS contain supermere markers and do not carry vesicle markers. Therefore, we can conclude that the fractions we obtained did indeed contain exomeres and supermeres, as the particle size and molecular composition were generally consistent with literature data on these particles. Furthermore, the obtained FCE and FCS fractions did not contain significant vesicle contamination, as the levels of vesicle markers in these fractions were quite low.

**Figure 2:**
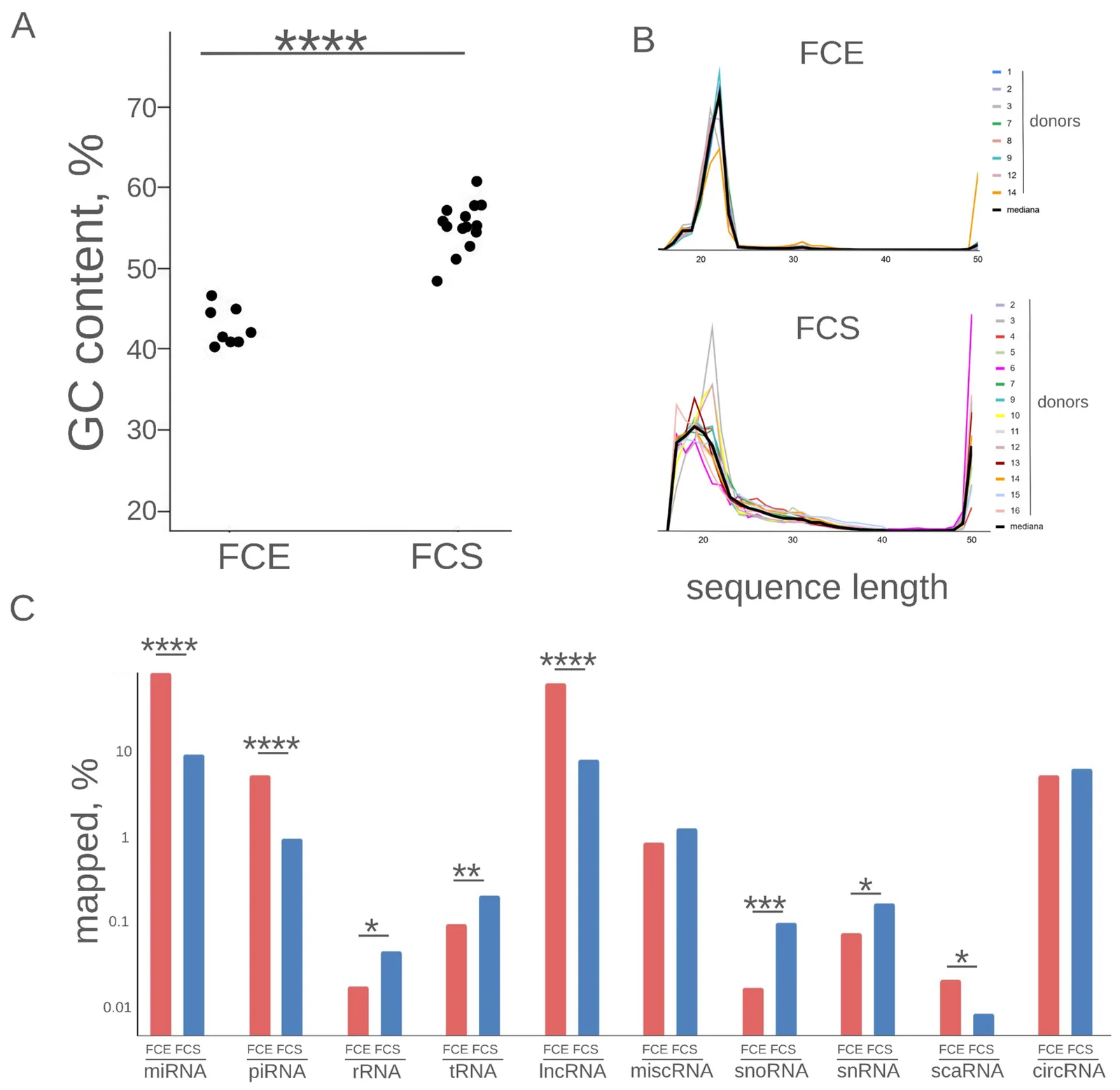
General characteristics of small RNA sequencing in FCE and FCS. (**A**) Comparison of the proportion of GC base pairs in small RNA sequencing from FCE and FCS. (**B**) Length of sequenced small RNA fragments from FCE and FCS. (**C**) Comparison of the percentage of mapped different types of non-coding RNAs in FCE and FCS. * 0.05 > *p*-value > 0.01; ** 0.01 > *p*-value > 0.001; *** 0.001 > *p*-value > 0.0001; **** 0.0001 > *p*-value.

Following isolation from FCE and FCS, RNA was extracted and subjected to high-throughput small RNA sequencing. Results regarding specific parameters of this sequencing are presented in Figure 2. As shown in Figure 2A, the GC content was significantly higher in FCS than in FCE. The distribution of fragment lengths also differed (Figure 2B); specifically, the fraction containing supermeres showed a substantially higher proportion of fragments in the 17–20 and 24–50 nucleotide ranges. Next, the sequenced sequences were classified by non-coding RNA type (Figure 2C), and their proportions in the obtained fractions were compared. As illustrated in the figure, the FCS fraction—compared to FCE—exhibited significantly lower percentages of miRNA, piRNA, lncRNA, and scaRNA, but higher percentages of rRNA, tRNA, snoRNA, and snRNA.

**Figure 3:**
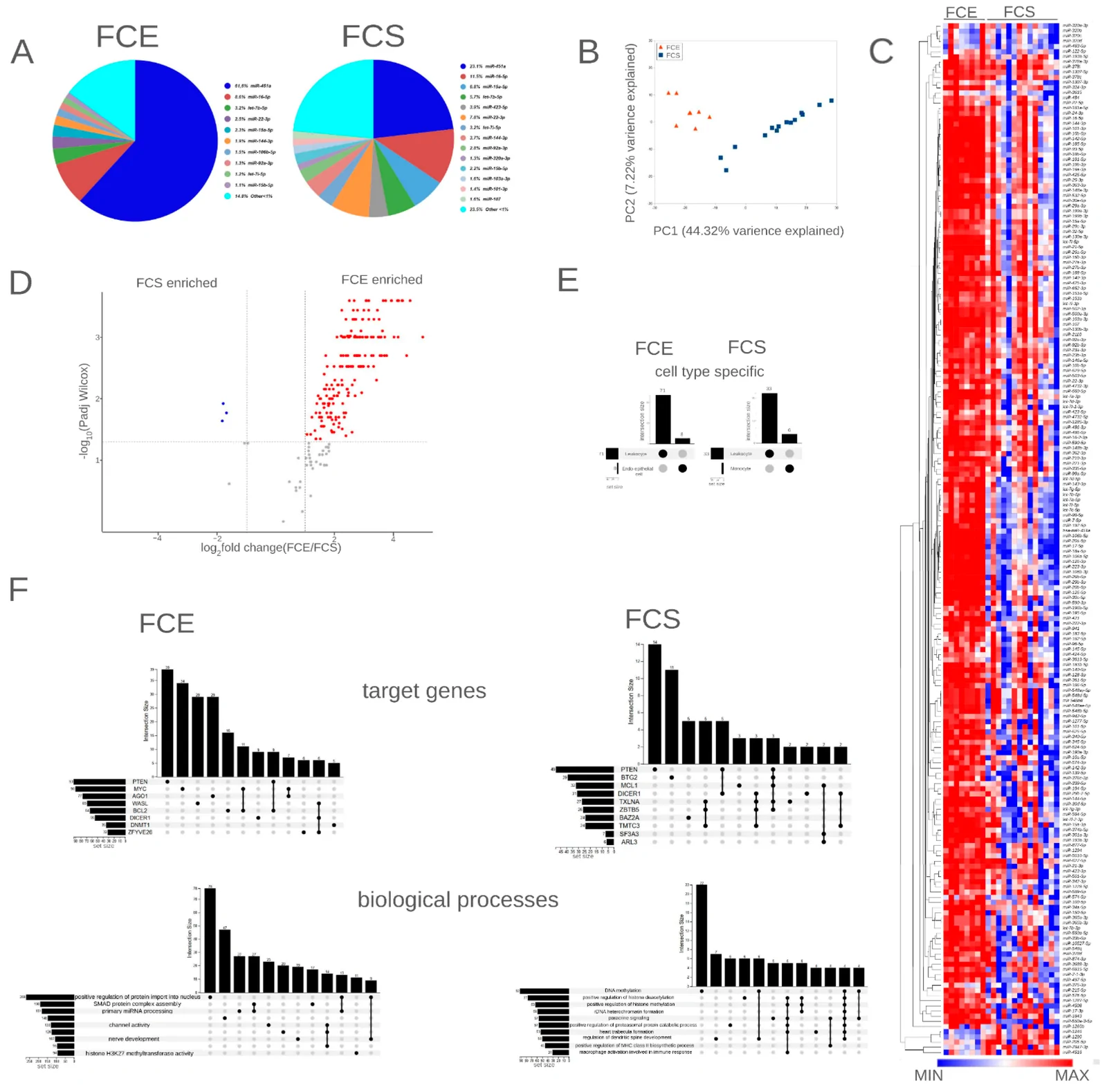
Comparison of the miRNA repertoire in FCE and FCS. (**A**). Plot of the levels of major miRNAs in FCE and FCS. The graph was generated based on the median RPM values for each fraction. (**B**) Analysis of the principal components of miRNA levels in FCE and FCS. (**C**) Heatmap of the 200 most highly expressed miRNAs in FCE and FCS. Clustering of miRNAs based on Pearson correlation. (**D**) Volcano plot comparing miRNA levels in FCE and FCS. (**E**) Upset plot of cells similar in composition to FCE and FCS, obtained as a result of miRNA enrichment analysis (MIEAA). (**F**) Upset plot of potential targets and biological processes (Gene Ontology), obtained as a result of miRNA enrichment analysis (MIEAA) from two fractions of FCE and FCS.

At the next stage, the composition of FCE and FCS miRNAs was compared (Figure 3). As can be seen in the presented figures, when applying the principal components method, FCE and FCS form quite distinct clusters (Figure 3B). The main miRNAs detected in the fractions are presented in Figure 3A. As can be seen in the presented figure, *miR-451a* constituted a significant part of the miRNA. It should also be noted that the majority of other highly expressed miRNAs (*miR-16-5p*, *let-7b-5p*, *miR-22-3p*, *miR-15a-5p*, *miR-144-3p*, *miR-92a-3p*, *let-7i-5p*, *miR-15b-5p*) coincided in the two fractions. A volcano plot (Figure 3D) and heat map (Figure 3C) were constructed based on the comparison of miRNA levels in the two fractions. Levels of 184 miRNAs were significantly higher (*p*-adj < 0.05, Fold change > 2) in the FCE fraction, and 3 miRNAs were significantly higher in the FCS fraction. The full list of miRNAs significantly different between the two fractions is presented in Table 1.

In addition, the study assessed the levels of novel miRNAs not present in MirBase (www.mirbase.org accessed on 27 February 2026). Only three novel miRNAs were found to be significantly more highly expressed in FCE compared to FCS. The sequences of these three RNAs, as well as the sequences of their precursors, are presented in Table 2. No novel miRNAs were found to be significantly more highly expressed in FCS compared to FCE.

Next, in silico miRNA enrichment analysis (MIEAA) was used to determine which cells the miRNA sets in the studied fractions were similar to (Figure 3E). As can be seen in the figure, FCE and FCS are most similar to leukocytes in their miRNA composition. Subsequently, the target genes and signaling pathways that the miRNA sets might involve were also studied in silico. FCE identified 4320 reliable (Qval < 0.05) genes in 326 signaling pathways regulated by miRNAs. For miRNAs expressed in FCS, 801 target genes and 235 signaling pathways were found. The most reliable pathways and genes are shown in Figure 3F.

**Figure 4:**
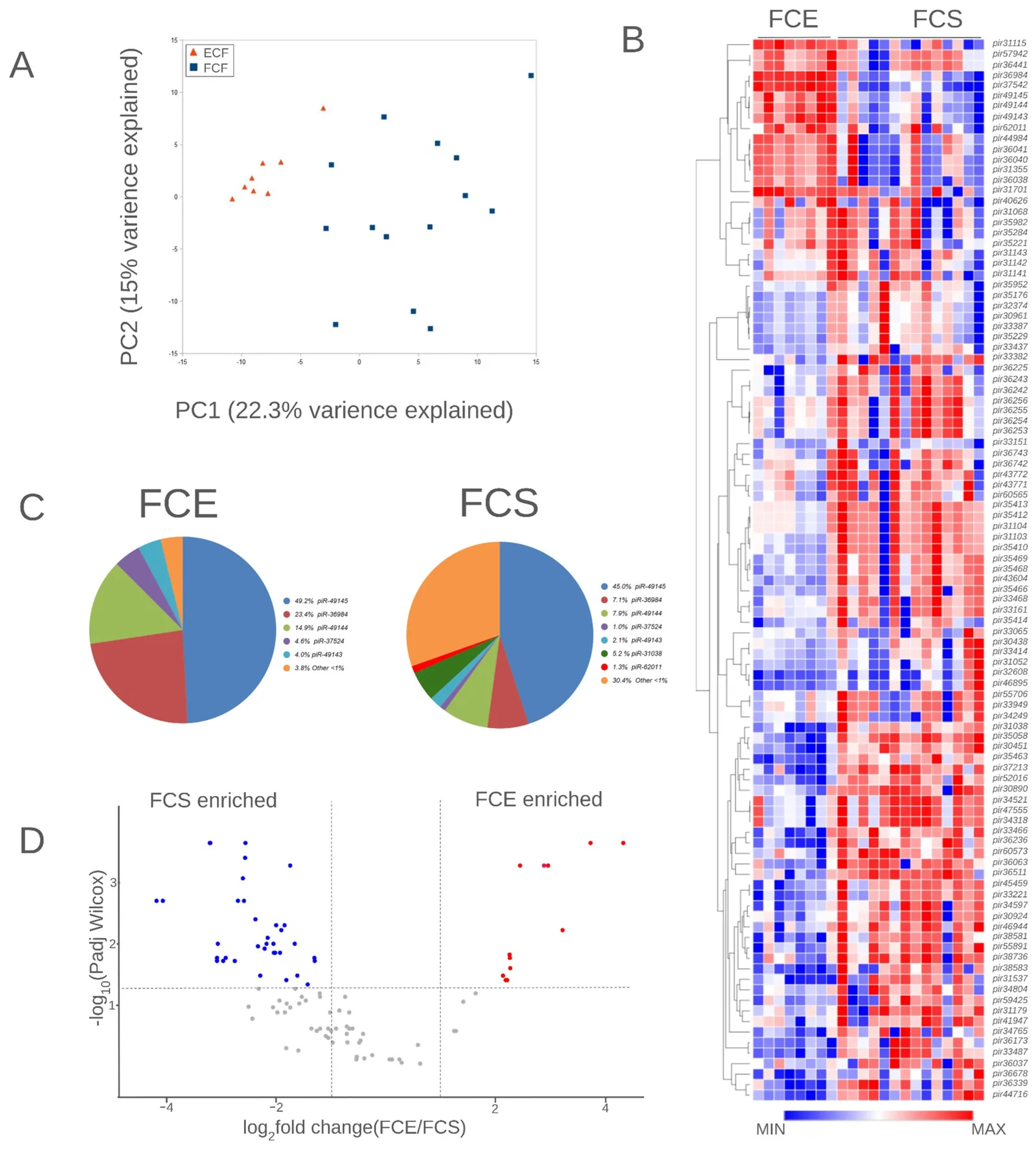
Comparison of piRNA repertoires in FCE and FCS. (**A**) Analysis of principal components of piRNA levels in FCE and FCS; (**B**) Heatmap of the 100 most highly expressed piRNAs in FCE and FCS. Clustering of miRNAs based on Pearson correlation; (**C**) Plot of major piRNA levels in FCE and FCS. The graph was generated based on the median RPM values for each fraction; (**D**) Volcano plot comparing piRNA levels in FCE and FCS.

The piRNA composition in the two obtained fractions was then analyzed. As can be seen in the figure (Figure 4C), *piR-49145* accounts for a significant (approximately 50%) portion of piRNA in FCE and FCS. The pattern of highly expressed piRNAs (*piR-36984, piR-49144, piR-37524, piR-49143*) is generally consistent in both fractions. It should also be noted that there is a significant difference in the piRNA composition in the two fractions, both when analyzed using the principal components method (Figure 4A), the heat map (Figure 4D), and the volcano diagram (Figure 4B). The level of 13 piRNAs was significantly higher in FCE and 34 in FCS (Figure 4C). The complete list of piRNAs whose levels significantly differed between the fractions is presented in Table 3.

## 3. Discussion

To compare the composition of exomeres and supermeres, it was necessary to obtain these particles. One method for isolating exomeres and supermeres is differential ultracentrifugation with preliminary separation of extracellular vesicles [4]. In some studies, serum albumin is first removed from blood plasma during isolation, since it precipitates with these particles during exomere and supermere isolation [4]. In others, such removal is not performed [11]. It should be mentioned that serum albumin itself can form complexes with nanoparticles [12] and its removal may probably somewhat distort the pattern of small RNAs contained in the fractions. Thus, although removing albumin from plasma results in purer preparations, it can also likely alter the pattern of small RNAs contained in exomeres and supermeres. For this reason, in this study, we chose to isolate exomeres and supermeres using ultracentrifugation but without removing serum albumin (Figure 1A).

Then, sequencing of the samples was carried out. After sequencing, the main parameters were assessed (Figure 2). One of the fundamental differences between the fractions was that the FCE (40–47%) had a lower proportion of GC than the FCS fraction (48–61%) (Figure 2A). This is probably due to the fact that the fraction contains more transcripts read from GC-rich regions of the genome. GC-rich RNAs include rRNA and tRNA [13], the percentage of which was significantly higher in FCS compared to FCE, whereas the levels of lncRNAs with a lower percentage of GC (36–42%) [14] were significantly lower in FCS compared to FCE (Figure 2C). If we compare our data (Figure 2C) on the distribution of different types of RNA between exomeres and supermeres obtained from the culture medium of DiFi cells with those presented in the work of Zhang et al. [4], we see that they coincide quite closely. This work showed that the levels of snoRNA, snRNA, and rRNA are higher in supermeres compared to exomeres, and the levels of miRNA and lncRNA are higher in exomeres compared to supermeres, which is completely consistent with our data. Separately, we would like to note that the work of Tosar et al. [15] showed that ribosomal proteins and long ribosomal RNAs are more abundant in exomeres compared to supermeres. In this paper, the authors suggest that the sequencing method significantly influences the rRNA ratio in the sequence. According to these authors, if small RNAs are separated, as was done in the work of Zhang et al. [4], rRNA is found to be more abundant in supermeres, while if longer and more complete sequences are examined, rRNA is found to be more abundant in exomeres. In our study, after library preparation, fragments up to 50 nucleotides in length were separated by gel electrophoresis, and only these fragments were sequenced. According to our data, rRNA levels are higher in FCS than in FCE (Figure 2C), which is consistent with the logic proposed by Tosar et al. [15].

Comparison of fragment length distribution shows that FCE contains a significantly higher proportion of transcripts 20–22 nucleotides long (Figure 2B). This is likely due to the higher proportion of miRNAs in this fraction compared to FCS. Moreover, the FCS fraction contains an increased proportion of both small transcripts 17–20 nucleotides long and larger sequences 24–50 nucleotides long. This may be due to the specific excision of fragments 17–20 and 24–50 nucleotides long from longer RNAs. This has been demonstrated most clearly for tRNAs, whose fragment sets vary greatly in different blood cell types [16], which may distinguish different levels of exomeres and supermeres. Other RNAs likely also produce short fragments that are specifically packaged into exomeres and supermeres, resulting in a specific fragment distribution pattern.

Further examination of our sequencing results (Figure 3A) revealed that *miR-451a* was the major miRNA in both fractions (more than half in FCE and almost a quarter in FCS). This miRNA is well known to be the major miRNA in erythrocytes [17], but it has also been shown to be expressed in leukocytes [18]. It is likely that some of these cells are the source of exomeres and supermeres. This miRNA is involved in the pathogenesis of many conditions, including various types of cancer [19,20,21], neurodegenerative disorders [22], heart [23] and liver disease [24].

Comparison of miRNA levels in fractions showed that, in FCS compared to FCE, only three miRNAs significantly predominate: *miR-4516*, *miR-1246*, and *miR-1290* (Figure 3D; Table 1). It was previously shown that *miR-4516* is the main miRNA in exomeres obtained by tangential flow filtration from the culture medium of HEK293T cells [9]. However, this study did not compare its levels in supermeres. Since the exomeres in this study were obtained using a different method and a different liquid, it cannot be said that our data contradict the work [9]. To date, exomeres and supermeres obtained by tangential flow filtration and ultracentrifugation have not been directly compared. Therefore, it cannot yet be determined whether the observed differences are attributable to the particle isolation method or to differences in the biological material used for fractionation. The function of *miR-4516* is known to be involved in the mechanisms of formation of many types of cancer. This is gastric [25], breast [26], liver [27], colorectal [28] and brain cancer [29].

It is known about *miR-1246* that it is found in supermeres obtained from the culture medium of DiFi cells [4]. It should be noted that this miRNA is one of the main RNAs of supermeres and exomeres [2], whereas in our fractions its level is not as high, and it is simply expressed significantly higher in FCS compared to FCE. At the same time, as mentioned above, *miR-451a* is the predominant miRNA in supermeres obtained from plasma, whereas *miR-1246* is not among the most abundant miRNAs in plasma-derived supermeres. This difference may be explained by the markedly different miRNA repertoire expressed by blood cells compared with DiFi cells, which may influence the composition of the supermeres they release. This effect may be particularly pronounced for *miR-451a*, which, as mentioned above, is the predominant miRNA in erythrocytes [17]. Nevertheless, *miR-1246* was still significantly more abundant in plasma-derived supermeres than in exomeres. The authors therefore hypothesize that *miR-1246* may represent a particularly reliable marker of supermeres, potentially being less dependent on the cellular source from which these particles are derived. *miR-1246* plays a role in different types of cancer [30,31,32], inflammatory bowel syndrome [33], infectious diseases [34,35]. *miR-1290* is also important in various types of cancer [36,37] in addition, cardiomyopathy [38] and autism spectrum disorders [39]. MiRNAs that are more highly prevalent in FCE compared to FCS are also quite important (Table 1). For example, *miR-20a-5p* (24-fold higher in FCE compared to FCS) plays an important role in cancer [40] as well as *miR-106a-5p* (20-fold higher in FCE) [41,42] and *miR-17-5p* (also 20-fold higher in FCE) [43].

It is not known with certainty which blood cells secrete supermeres and exomeres into the blood. However, using miRNA enrichment algorithms, it is possible to attempt to predict in silico how similar an miRNA set is to the miRNA patterns of different cell types. As shown in Figure 3E shows that miRNAs in FCE are similar in composition to leukocytes and endothelial cells, while FCS are similar to leukocytes and, more specifically, monocytes. It is interesting to note that a significant portion (approximately 35%) of extracellular plasma vesicles is secreted by erythrocytes and platelets [44]. However, enrichment analysis of our samples did not reveal a reliable miRNA pattern characteristic of these cells. This may be due to the fact that exomeres and supermeres are expressed at a much lower level by these cells. However, the limitations of this approach to identifying particle-producing cells should be considered, as the miRNA pattern in the cells and in the particles they produce may likely differ significantly.

Subsequently, in silico analysis was used to determine which target genes are most likely affected by miRNAs in exomeres and supermeres. As can be seen in Figure 3D the most important protein that, according to these data, is regulated by miRNAs from both fractions studied is the protein and lipid phosphatase PTEN [45]. Proteins associated with the functioning of the miRNA system itself, DICER1 and AGO1 [46], are also likely to be negatively regulated. In addition, the products of the BCL-2 genes and its family member MCL1 control apoptosis [47]. MiRNAs in these fractions also influence the transcription factors MYC, ZBTB5, and the transcription factor regulator BTG2. Furthermore, they influence the chromatin structure regulator BAZ2A, DNA methyltransferase-1 (DNMT1), and the spliceosome component SF3A3, which is responsible for intron excision. Regulation of the TXLNA gene encoding Interleukin-14 is likely related to blood cell function.

Focusing on some of the biological processes regulated by the miRNA repertoire from the fractions, it turns out that these molecules highly likely influence the functioning of the cell nucleus. These include histone deacetylation and methylation, DNA methylation, heterochromatin formation, and protein transport into the nucleus. Furthermore, miRNAs likely influence embryonic processes of nerve development and the formation of cardiac trabeculae. Enrichment analysis also reveals a link with immune system processes, such as macrophage activation and MHCII synthesis.

However, it should be noted that the in silico analysis is only predictive in nature and cannot provide direct evidence of target gene suppression, signaling pathway regulation, or identification of the cellular sources of the particles. Definitive confirmation would require appropriate experimental validation, including functional assays to assess the effects of the identified miRNAs on their target genes and signaling pathways, as well as experimental approaches to determine the cellular origin of the particles.

Determination of piRNA levels revealed that a significant portion of these molecules in both fractions is *pir-49145* (Figure 4). This piRNA plays a role in gastric [48], head and neck cancer [49]. It should be mentioned that three piRNAs presented in this panel (*pir-49145*, *pir-49144*, *pir-49143*) were previously detected in the major RNAs of erythrocytes and leukocytes [50].

It is likely that exomeres and supermeres with these piRNAs are secreted by these cells. If we consider the role of piRNAs that have the most significant differences in levels in the studied fractions, it turns out that *piR-45459*, the concentration of which is 18 times higher in FCS (Table 3), is suppressed in erythrocytes with a severe course of COVID [50], and *piR-37524* piRNA, which is 20 times more present in FCE (Table 3), is overexpressed in colorectal cancer cells [51].

Comparing the levels of piRNA and miRNA in the fractions, it is interesting to note that the majority of miRNAs are highly prevalent in the FCE fraction (184 in FCE, 3 in FCS), while piRNA is much less prevalent in FCE (13 in FCE, 34 in FCS). It is likely that the signaling function of piRNA is more important for supermeres than miRNA. How exactly piRNAs work in supermeres is unclear. It is possible that piRNAs in these particles are somehow related to the functioning of the immune system [52]. Perhaps the role of these particles is similar to the role of piRNAs in vesicles, which have been shown to be secreted by macrophages [53].

When considering the novelty of our study as a whole, it is important to emphasize that the molecular composition of exomeres and supermeres from blood plasma has not yet been comprehensively investigated. The preparations obtained in the present study cannot be considered completely pure, as albumin was not removed from the samples prior to the centrifugation steps. Nevertheless, the data obtained on the molecular composition of fractions containing exomeres and supermeres are, to the best of our knowledge, unique. The criteria used for donor selection are also particularly important. It is well known that the composition of small RNAs in plasma or vesicles depends on the age of the donors [54]. It is also important to note that older age groups are much more likely to exhibit various diseases that can be diagnosed using the small RNAs contained in these particles. For this reason, to ensure greater homogeneity in the group, we decided to use only donors within a narrow age group of 63–72 years. The decision to include donors within this age range was also based on the consideration that characterization of the small RNA composition of particles in a healthy control group may, in the future, provide a reference for comparison with small RNA patterns observed in patients with diseases that occur more frequently in older individuals.

To summarize our work, it should be noted that although the pattern of small RNAs most highly expressed in the fractions is fairly similar, the levels of most RNAs differ significantly. This is evident from most of the presented diagrams (volcano plots, principal component analysis, GC composition, fragment length analysis). One possibility, albeit a highly speculative one, is that this difference may be related, at least in part, to potentially distinct signaling functions of the two particle pools.

## 4. Materials and Methods

### 4.1. Donors and Blood Collection

Blood (5 mL) was collected from the antecubital vein of healthy donors (8 men and 8 women, aged 63–72 years) after informed consent was obtained. Clinical data on the donors are presented in Table 4. The only inclusion criterion was the donors’ age, while the exclusion criterion was the presence of any acute or chronic disease. Platelet-poor plasma was obtained from the blood via sequential centrifugation at 1500× *g*, 3000× *g*, and 3000× *g* for 10 min each, with the supernatant transferred to a new tube at each step. The supernatant from the final centrifugation (platelet-poor plasma) was used for the subsequent separation of vesicles and the isolation of fractions containing exomeres and supermeres.

### 4.2. Fraction Isolation

The procedure for separating plasma fractions is shown in Figure 1A. Samples were centrifuged in batches of four in a randomized order. All ultracentrifugation steps were performed using an Optima XPN-100 ultracentrifuge equipped with a fixed-angle rotor (Type 70 Ti, k-factor 31) and 27 mL tubes. Samples were brought to a final volume of 27 mL with phosphate-buffered saline (PBS). Two milliliters of plasma were centrifuged for 4 h at 170,000× *g* to pellet the vesicles (vesicle-containing fraction). This fraction was used solely for immunoblotting. The post-centrifugation supernatant was transferred to a new tube and centrifuged for 20 h at 170,000× *g*. The pellet (fraction containing exomeres) was washed by repeated centrifugation (20 h at 170,000× *g*) and used for RNA isolation, dynamic light scattering, scanning electron microscopy, immunoblotting, and small RNA sequencing. The supernatant was centrifuged at 370,000× *g* for 20 h. The pellet (fraction containing supermeres) was washed by repeated centrifugation (20 h at 370,000× *g*) and also used for RNA isolation, dynamic light scattering, scanning electron microscopy, immunoblotting, and small RNA sequencing. The fractions were washed once by resuspending the pellet (approximately 500 μL) in phosphate-buffered saline (PBS) to a final volume of 27 mL, followed by repeat centrifugation at the corresponding speed and removal of the supernatant.

### 4.3. Low-Voltage Scanning Electron Microscopy

Supermere and exomere fractions were fixed on glass slides using a previously described method [55]. Observations were performed using a Zeiss Auriga microscope at an accelerating voltage of 400–600 V.

### 4.4. Immunoblotting

FCV, FCE, and FCS samples were normalized for total protein concentration using the RC DC protein assay (Bio-Rad, Hercules, CA, USA) ), incubated for 10 min with Laemmli buffer, and loaded onto a 7–15% polyacrylamide gel. Following electrophoresis, proteins were transferred to a nitrocellulose membrane and stained with Ponceau S and specific antibodies against Alix (Cell Signaling Technology; 2171, Danvers, MA, USA ), GM130 (Cell Signaling Technology; 12480), GPC1 (Cloud-Clone Corp; PAB032Hu01, Wuhan, China), ICAM (Cell Signaling Technology; 67836), and TSG101 (Cloud-Clone Corp; MAC814Hu21), followed by staining with horseradish peroxidase-conjugated secondary antibodies (Bio-Rad). Signal detection was carried out using the Femto SuperSignal reagent (Thermo Fisher Scientific, Waltham, MA, USA). The fluorescent signal was detected using a ChemiDoc instrument (Bio-Rad).

### 4.5. Dynamic Light Scattering

The size of particles contained in the exomere and supermere fractions was evaluated using DLS on a Photocor Complex instrument (Photocor, Moscow, Russia), following a previously described procedure [56]. Measurements were performed over a period of 10 min at a 90° angle, a temperature of 20 °C, and a laser wavelength of 455 nm. The images were processed using the DynaLS software (v2.x).

### 4.6. RNA Isolation, Small RNA Library Preparation and sRNA Sequencing

RNA extraction and library preparation were performed simultaneously for all samples. Exomere and supermere fraction samples were mixed with Trizol LS reagent (Thermo Fisher Scientific), and RNA was isolated according to the manufacturer’s protocol. Following isolation, the samples were resuspended in 7 µL of RNase-free deionized water (Evrogene, Moscow, Russia). RNA concentration was measured using the QuantiFluor^®^ RNA System (Promega, Madison, WI, USA) on a Fluo-200 instrument (Allsheng, Hangzhou, China).

To construct the libraries, 10–20 ng of RNA was used. Small RNA libraries were prepared using the MGIEasy Small RNA Library Prep Kit V2.0 (BGI-940-000196-00, BGI, Beijing, China). The libraries were purified via 6% polyacrylamide gel electrophoresis by excising the 100–135 bp band, which corresponds to fragment lengths of 15–50 bp. Small RNA sequencing was performed on a BGI DNBSEQ-G400 platform. Sequencing was performed using the DNBSEQ-G400 High-throughput Sequencing Set (SM Small RNA FCL SE50, BGI, Beijing, China). All samples included in the study were sequenced on a single lane. A total of 520 million reads were obtained.

### 4.7. Bioinformatics and Statistics

120–2000 MB of data with Q30 scores of 95–98 for each sample were uploaded to the Mirmaster service (version 2.0.0) [57]. Mirmaster was used to perform adapter trimming, assess key sequencing parameters, annotate RNA, count reads, calculate fold change and adjusted Wilcoxon *p*-values, predict novel miRNAs, and conduct principal component analysis. RNA annotation was performed using the miRBase v. 22.1 [58], RNAcentral v. 15 [59], Ensembl ncRNA v. 100 [60], and circBase databases [61] (accessed 25 October 2020). Heatmaps were generated from log-transformed data using the phantasus v1.31.1 software [62]. The 200 most highly expressed miRNAs and 100 piRNAs were used to generate heatmaps. Clustering of miRNAs and piRNAs was performed based on the Pearson correlation. Significant differences were determined using the Wilcoxon test; differences were considered significant at *p*-adj < 0.05 and a fold change > 2. Additionally, for the construction of volcano plots and level comparisons, only those miRNAs and piRNAs with a median read count exceeding 10 RPM in at least one of the fractions (FCE or FCS) were included. The MIEAA 2.0 service was used for miRNA enrichment analysis and plot generation [63]. Only data points with a Q-value < 0.05 were used to generate the plots. Data were loaded automatically from Mirmaster lists, with miRNAs sorted by descending median RPM. The cell type that FCS or FCE resembled in terms of miRNA composition was determined using the Human Cellular microRNAome database [64]. Potential target proteins were identified using the MirTarBase V9.0 database [65]. This database was also used to identify potential biological processes and cellular components, which were determined using Gene Ontology [66]. Raw read count data for each miRNA and piRNA are provided in the Supplementary Materials. The Supplementary Materials also include a list of Gene Ontology terms and target proteins identified through enrichment analysis.

## 5. Conclusions

The authors consider the main outcome of the present study to be the characterization of the miRNA and piRNA composition of fractions containing exomeres and supermeres isolated from blood plasma. To the best of our knowledge, such an analysis has not previously been performed for plasma-derived exomere- and supermere-containing fractions. Although the most abundant miRNAs and piRNAs were largely similar between the two fractions, their overall small RNA profiles showed substantial differences. The in silico analyses performed using MIEAA in the present study are predictive in nature and should therefore be interpreted with caution.

## 6. Limitations

Several limitations of the present study should be noted.

1. The differences in small RNA profiles identified in this study apply specifically to fractions obtained from human blood plasma by differential ultracentrifugation. Fractions obtained from other biological fluids, such as cell culture media, urine, or saliva, as well as fractions isolated using other methods, such as asymmetric flow field-flow fractionation, may have different small RNA compositions.
2. The present study characterized the small RNA composition of plasma-derived fractions only in older individuals. The composition of small RNAs in similar fractions may differ substantially in other age groups.
3. The fractionation protocol used in this study did not include prior removal of albumin from the blood plasma. The authors cannot exclude the possibility that removal of albumin before fractionation using an otherwise comparable protocol could result in more pronounced differences in the composition of these fractions.

## Figures and Tables

**Table 1:** List of miRNAs, the expression of which significantly differs in FCE and FCS.

miRNA	Fold Change	*p*-Value Adj	Overexpressed in Fraction
*miR-4516*	3.5	0.023	FCS
*miR-1290*	3.5	0.012	FCS
*miR-1246*	3.2	0.017	FCS
*miR-93-5p*	5.7	0.000257	FCE
*miR-7-5p*	6.4	0.000257	FCE
*miR-144-5p*	8.4	0.000257	FCE
*miR-18b-5p*	8.5	0.000257	FCE
*miR-423-3p*	9	0.000257	FCE
*miR-223-3p*	12.6	0.000257	FCE
*miR-106b-5p*	13.3	0.000257	FCE
*miR-126-3p*	15.2	0.000257	FCE
*miR-106b-3p*	15.3	0.000257	FCE
*miR-451a*	17.6	0.000257	FCE
*miR-20b-5p*	18.1	0.000257	FCE
*miR-18a-5p*	19.6	0.000257	FCE
*miR-17-5p*	19.8	0.000257	FCE
*miR-106a-5p*	19.9	0.000257	FCE
*miR-20a-5p*	23.8	0.000257	FCE
*miR-196b-5p*	4.7	0.000368	FCE
*miR-192-5p*	6.2	0.000368	FCE
*let-7c-5p*	6.9	0.000368	FCE
*miR-34a-5p*	8	0.000368	FCE
*miR-532-5p*	8.9	0.000368	FCE
*miR-98-5p*	11.0	0.000368	FCE
*let-7b-5p*	5.2	0.000515	FCE
*miR-99a-5p*	6.2	0.000515	FCE
*let-7g-5p*	6.4	0.000515	FCE
*miR-16-5p*	6.6	0.000515	FCE
*let-7a-5p*	7.7	0.000515	FCE
*let-7f-5p*	7.8	0.000515	FCE
*miR-324-3p*	9	0.000515	FCE
*miR-941*	10.1	0.000515	FCE
*miR-16-2-3p*	11.4	0.000515	FCE
*miR-100-5p*	5.9	0.000795	FCE
*miR-29b-3p*	7.4	0.000795	FCE
*miR-30e-5p*	12.4	0.000795	FCE
*miR-425-5p*	4.7	0.000965	FCE
*miR-486-3p*	5,4	0.000965	FCE
*let-7d-5p*	5.7	0.000965	FCE
*miR-199b-3p*	5.8	0.000965	FCE
*miR-199a-3p*	5.8	0.000965	FCE
*miR-143-3p*	6.5	0.000965	FCE
*miR-598-3p*	4.2	0.001	FCE
*miR-142-3p*	4.9	0.001	FCE
*miR-185-5p*	5.0	0.001	FCE
*miR-425-3p*	5.1	0.001	FCE
*miR-92b-3p*	5.5	0.001	FCE
*miR-150-5p*	5.5	0.001	FCE
*miR-181c-5p*	5.6	0.001	FCE
*miR-194-5p*	6.4	0.001	FCE
*miR-148a-3p*	6.6	0.001	FCE
*miR-17-3p*	6.9	0.001	FCE
*miR-28-5p*	7.3	0.001	FCE
*miR-19b-3p*	7.3	0.001	FCE
*miR-342-3p*	7.7	0.001	FCE
*miR-139-3p*	8.3	0.001	FCE
*miR-340-5p*	8.6	0.001	FCE
*miR-126-5p*	9.5	0.001	FCE
*miR-30b-5p*	10.0	0.001	FCE
*miR-7-1-3p*	11.2	0.001	FCE
*miR-374a-5p*	11.6	0.001	FCE
*miR-33a-5p*	12.0	0.001	FCE
*miR-324-5p*	13.1	0.001	FCE
*miR-151a-3p*	16.2	0.001	FCE
*miR-30a-5p*	17.9	0.001	FCE
*miR-374b-5p*	32.0	0.001	FCE
*miR-181a-5p*	3.2	0.002	FCE
*let-7a-3p*	4.6	0.002	FCE
*miR-29a-3p*	4.9	0.002	FCE
*miR-5010-5p*	5.2	0.002	FCE
*miR-574-3p*	5.5	0.002	FCE
*miR-590-3p*	6.4	0.002	FCE
*miR-26b-5p*	6.6	0.002	FCE
*miR-142-5p*	6.7	0.002	FCE
*miR-19a-3p*	6.9	0.002	FCE
*miR-409-3p*	7.0	0.002	FCE
*miR-210-3p*	7.2	0.002	FCE
*let-7b-3p*	8.2	0.002	FCE
*miR-139-5p*	9.6	0.002	FCE
*miR-30c-5p*	10.0	0.002	FCE
*miR-1228-5p*	11.7	0.002	FCE
*let-7d-3p*	12.1	0.002	FCE
*miR-625-5p*	13.0	0.002	FCE
*miR-30d-5p*	13.6	0.002	FCE
*miR-301a-3p*	21.8	0.002	FCE
*miR-1307-3p*	3.4	0.003	FCE
*miR-22-3p*	4.0	0.003	FCE
*miR-21-5p*	4.6	0.003	FCE
*let-7e-5p*	4.8	0.003	FCE
*miR-101-3p*	5.3	0.003	FCE
*miR-101-5p*	5.5	0.003	FCE
*miR-140-5p*	5.6	0.003	FCE
*miR-345-5p*	5.9	0.003	FCE
*miR-486-5p*	6.1	0.003	FCE
*miR-99b-5p*	6.1	0.003	FCE
*miR-550a-5p*	6.1	0.003	FCE
*miR-421*	7.1	0.003	FCE
*miR-222-3p*	7.3	0.003	FCE
*miR-190a-3p*	7.4	0.003	FCE
*miR-365a-3p*	7.5	0.003	FCE
*miR-365b-3p*	7.5	0.003	FCE
*miR-376c-3p*	7.8	0.003	FCE
*miR-497-5p*	7.9	0.003	FCE
*miR-339-3p*	8.9	0.003	FCE
*miR-1277-5p*	10.0	0.003	FCE
*miR-181b-5p*	3.1	0.004	FCE
*miR-92a-3p*	4.0	0.005	FCE
*miR-28-3p*	4.7	0.005	FCE
*miR-33b-5p*	6.4	0.005	FCE
*let-7g-3p*	10.8	0.005	FCE
*miR-502-3p*	3.3	0.006	FCE
*miR-26a-5p*	3.7	0.006	FCE
*miR-195-5p*	4.3	0.006	FCE
*miR-361-5p*	4.4	0.006	FCE
*miR-193a-3p*	4.8	0.006	FCE
*miR-140-3p*	4.8	0.006	FCE
*miR-186-5p*	6.1	0.006	FCE
*miR-221-3p*	3.1	0.007	FCE
*miR-188-5p*	3.2	0.007	FCE
*miR-191-5p*	3.5	0.007	FCE
*miR-378d*	3.9	0.007	FCE
*miR-1307-5p*	5.3	0.007	FCE
*miR-10a-5p*	5.4	0.007	FCE
*miR-584-5p*	4.4	0.008	FCE
*miR-206*	5.6	0.008	FCE
*let-7f-1-3p*	7.6	0.008	FCE
*miR-24-3p*	2.7	0.009	FCE
*miR-660-5p*	3.3	0.009	FCE
*miR-25-3p*	3.9	0.009	FCE
*miR-148b-3p*	4.2	0.009	FCE
*miR-3200-3p*	4.3	0.009	FCE
*miR-624-5p*	5.5	0.009	FCE
*miR-369-5p*	5.5	0.009	FCE
*miR-744-5p*	6.5	0.009	FCE
*miR-23a-3p*	2.8	0.01	FCE
*let-7i-5p*	2.9	0.01	FCE
*miR-363-3p*	3.5	0.01	FCE
*miR-130a-3p*	3.7	0.01	FCE
*miR-877-5p*	3.8	0.01	FCE
*miR-574-5p*	2.6	0.012	FCE
*miR-146a-5p*	3.1	0.012	FCE
*miR-27a-3p*	3.1	0.012	FCE
*miR-942-5p*	3.5	0.012	FCE
*miR-15b-3p*	3.5	0.012	FCE
*miR-4732-3p*	3.9	0.012	FCE
*miR-15b-5p*	4.8	0.012	FCE
*miR-576-5p*	3.5	0.013	FCE
*miR-27b-3p*	3.1	0.014	FCE
*miR-378a-3p*	2.6	0.017	FCE
*miR-378c*	2.8	0.017	FCE
*miR-1294*	3.3	0.017	FCE
*miR-128-3p*	3.6	0.017	FCE
*let-7i-3p*	3.7	0.017	FCE
*let-7f-2-3p*	5.0	0.017	FCE
*miR-144-3p*	5.2	0.017	FCE
*miR-1843*	2.3	0.02	FCE
*miR-29c-3p*	2.8	0.02	FCE
*miR-23b-3p*	2.9	0.02	FCE
*miR-500a-3p*	2.9	0.02	FCE
*miR-652-3p*	3.0	0.02	FCE
*miR-2110*	3.4	0.02	FCE
*miR-107*	3.6	0.02	FCE
*miR-103a-3p*	3.6	0.02	FCE
*miR-193b-3p*	4.1	0.02	FCE
*miR-10b-5p*	4.4	0.02	FCE
*miR-550a-3-5p*	5.0	0.02	FCE
*miR-505-3p*	3.8	0.022	FCE
*miR-342-5p*	4.1	0.022	FCE
*miR-29b-2-5p*	2.9	0.023	FCE
*miR-874-3p*	4.5	0.025	FCE
*miR-628-3p*	3.9	0.026	FCE
*miR-3688-3p*	2.9	0.028	FCE
*miR-15a-5p*	3.1	0.028	FCE
*miR-182-5p*	3.1	0.028	FCE
*miR-6815-5p*	4.4	0.029	FCE
*miR-215-5p*	4.8	0.031	FCE
*miR-130b-3p*	2.5	0.033	FCE
*miR-501-3p*	3.7	0.033	FCE
*miR-10527-5p*	3.8	0.035	FCE
*miR-15a-3p*	3.9	0.035	FCE
*miR-4508*	6.0	0.035	FCE
*miR-629-5p*	2.1	0.038	FCE
*miR-1287-5p*	2.3	0.038	FCE
*miR-548q*	5.2	0.04	FCE
*miR-151a-5p*	2.6	0.045	FCE
*miR-32-5p*	2.6	0.045	FCE
*miR-484*	2.9	0.045	FCE

**Table 2:** Predicted novel miRNAs that overexpressed in FCE.

miRNA	Fold Change	*p*-Value Adj	miRNA, Nucleotide Sequence	Pre-miRNA, Nucleotide Sequence
*pred-nov-miR-369-5p*	3.9	0.022	AUAGUGUGUAUGUGUGUGUGU	AUAGUGUGUAUGUGUGUGUGUGUGUAUAUAUAUACACACACACAUACACACUAUAC
*pred-nov-miR-604-5p*	6.5	0.003	UGAGAGGCAGAGGUUGCAGUGG	UGAGAGGCAGAGGUUGCAGUGGCACGAUCUCAGGUCACUGCAACCUCUGCCUCCG
*pred-nov-miR-2-3p*	9.8	0.002	GGGUGCGGGCCGGCGGGGUC	GACCUCGCCGUCCCGCCCGCCGCCUUCUGCGUCGCGGGUGCGGGCCGGCGGGGUC

**Table 3:** List of piRNAs with significant differences in expression levels between the FCE and FCS fractions.

piRNA	Fold Change	*p*-Value Adj	Overexpressed in Fraction
*piR-45459*	18.2	0.002	FCS
*piR-33221*	16.8	0.002	FCS
*piR-35058*	9.3	0.000229	FCS
*piR-30451*	9.2	0.000229	FCS
*piR-34597*	8.5	0.017	FCS
*piR-38581*	8.5	0.019	FCS
*piR-55891*	8.4	0.01	FCS
*piR-30924*	7.8	0.019	FCS
*piR-46944*	7.6	0.017	FCS
*piR-38736*	6.8	0.019	FCS
*piR-36511*	6.5	0.002	FCS
*piR-52016*	6.1	0.000862	FCS
*piR-37213*	6.0	0.002	FCS
*piR-35463*	5.9	0.0004	FCS
*piR-35462*	5.9	0.000229	FCS
*piR-36236*	5.2	0.004	FCS
*piR-31179*	4.9	0.033	FCS
*piR-43604*	4.6	0.012	FCS
*piR-60573*	4.5	0.01	FCS
*piR-36063*	4.5	0.008	FCS
*piR-33466*	4.1	0.01	FCS
*piR-35467*	4.1	0.014	FCS
*piR-35468*	4.1	0.014	FCS
*piR-35410*	4.0	0.005	FCS
*piR-35469*	3.8	0.014	FCS
*piR-30890*	3.7	0.006	FCS
*piR-35411*	3.6	0.005	FCS
*piR-36339*	3.5	0.039	FCS
*piR-31038*	3.4	0.000534	FCS
*piR-33468*	3.1	0.033	FCS
*piR-33161*	2.7	0.046	FCS
*piR-35413*	2.5	0.017	FCS
*piR-31104*	2.5	0.019	FCS
*piR-35412*	2.5	0.019	FCS
*piR-37524*	20.2	0.000229	FCE
*piR-36984*	13.3	0.000229	FCE
*piR-31115*	9.4	0.006	FCE
*piR-49143*	7.8	0.000534	FCE
*piR-49144*	7.4	0.000534	FCE
*piR-49145*	5.5	0.000534	FCE
*piR-36040*	4.8	0.025	FCE
*piR-31355*	4.8	0.025	FCE
*piR-36039*	4.8	0.017	FCE
*piR-31701*	4.8	0.015	FCE
*piR-36041*	4.6	0.039	FCE
*piR-44984*	4.6	0.039	FCE
*piR-36038*	4.4	0.033	FCE

**Table 4:** Demographic data on the sex and age of the donors whose blood was used in the study. “X” indicates that sequencing of the corresponding fraction was successfully performed for a given donor. “-“ indicates that sequencing of the corresponding fraction could not be performed due to insufficient amounts of RNA, cDNA, or the final library. Individual donor sequencing results are provided in the Supplementary Materials.

Donor Number	Sex	Age, Years	FCE	FCS
1	female	72	X	-
2	male	71	X	X
3	male	71	X	X
4	male	70	-	X
5	male	69	-	X
6	male	68	-	X
7	female	67	X	X
8	male	67	X	-
9	male	66	X	X
10	male	66	-	X
11	female	64	-	X
12	female	65	X	X
13	female	65	-	X
14	female	65	X	X
15	female	63	-	X
16	female	63	-	X

## Data Availability

The original contributions presented in this study are included in the article/Supplementary Materials. Further inquiries can be directed to the corresponding author.

## Supplementary Materials

The following supporting information can be downloaded at: https://www.mdpi.com/article/10.3390/ijms27209268/s1.
